# Beneath the Surface: Aquatic Models Illuminate Host–Microbe Interactions

**DOI:** 10.3390/biology15181603

**Published:** 2026-09-11

**Authors:** Kathryn B. Lorbacher, Tram Le, Morgan E. Milton, Karen Mruk

**Affiliations:** 1 Department of Pharmacology and Toxicology, Brody School of Medicine, East Carolina University, Greenville, NC 27834, USA; brookhartk23@students.ecu.edu; 2 Department of Biochemistry and Molecular Biology, Brody School of Medicine, East Carolina University, Greenville, NC 27834, USA; letr16@students.ecu.edu

**Keywords:** aquatic models, conditional pathogenicity, *Vibrio*, zebrafish

## Abstract

Microbial outcomes emerge from dynamic interactions among host defenses, microbial traits, and environmental context. This narrative review examines aquatic host–microbe systems, with emphasis on zebrafish and *Vibrio* species, as experimentally tractable aquatic models for understanding how interactions shift across a spectrum from mutualism to pathogenesis. We first define major aquatic invertebrate and vertebrate models, highlighting how each balances scalability, genetic accessibility, physiological complexity, and translational relevance. We then use *Vibrio* species as a framework for moving beyond the pathogen versus non-pathogen classification, emphasizing conditional pathogenicity, host-dependent outcomes, and shared molecular toolkits between symbionts and pathogens. Finally, we highlight host responses including barriers and immunity. Aquatic models reveal that disease results from context-dependent regulation of both host and microbial programs. By integrating the diversity of aquatic models, aquatic host–microbe research presents a unique opportunity to define the continuum of outcomes from mutualism to pathogenesis.

## Simple Summary

Aquatic animals provide valuable systems for studying how host, microbial, and environmental factors shape infection outcomes. Invertebrates offer simple, low-cost models, while vertebrates add greater physiological and genetic relevance. Together, these animal models highlight that microbes are not simply pathogens or symbionts, but rather display a spectrum of traits that are dependent on the animal, environment, microbe, and immune response. *Vibrio* species illustrate this clearly, as bacteria in the genus can act as beneficial partners, harmless colonizers, or disease-causing pathogens in different contexts. Host defenses, including barriers, the gut, the blood–brain barrier, neutrophils, and macrophages, ultimately help determine whether infection leads to disease, tolerance, or clearance. In this narrative review, we highlight that aquatic models reveal that infection outcomes are not predetermined but rather can change based on dynamic interactions among the host, microbe, and environment.

## 1. Introduction

Microbial behavior is rarely fixed. Behavioral plasticity of microbes was first described in a 1976 study where bacteria had different swimming patterns that persisted over time despite controlled conditions and genetically identical backgrounds [1]. Since that seminal work, scientists have begun to understand strategies that microbes have developed, such as bet-hedging, to respond to environmental changes [2]. Despite major advances in determining how bacterial behavior changes in response to the environment, whether a microbe causes disease or forms a beneficial association based on the host it encounters is still not well understood. This context dependence is particularly important in aquatic environments, where hosts are continuously exposed to diverse microbial communities [3]. These communities include aquatic organisms that have evolved layered defenses, including physical barriers, physiological barriers, resident microbiota, and innate immune cells. At the same time, microbes have evolved strategies to colonize, compete, evade, or exploit those defenses. Together, these opposing pathways make aquatic systems powerful models for understanding how microbial plasticity changes in response to a variety of factors.

Aquatic models provide powerful systems for dissecting host–microbe interactions because they span a range of biological and physiological complexity while preserving experimental tractability. Less complex invertebrate models such as brine shrimp are low cost and scalable, allowing researchers to examine conserved innate protective mechanisms. In contrast, vertebrate aquatic models provide physiological and genetic similarity to mammalian systems while retaining key advantages observed in invertebrates. Zebrafish have become especially important for microbial research because transparent embryos and larvae enable direct visualization of infection dynamics, immune cell recruitment, gut colonization, and host tissue responses.

In this review, we highlight aquatic host–microbe systems, using *Vibrio* species and zebrafish as a comparative framework, to examine how outcomes emerge from the interaction between host defenses, microbial determinants, and environmental context. Several bacterial species discussed have undergone taxonomic revision and are no longer within the *Vibrio* genus. Despite reclassification, many of the genetic and physiological traits discussed in this review are shared across species. Therefore, we use the term “*Vibrio*” to refer to all *Vibrio* and *Vibrio*-like species. We first define common aquatic models and explain how they are used to study host–pathogen biology. We compare aquatic models across a biological complexity, helping readers understand which aquatic model may be suited to answering specific host–pathogen questions. We then explain how microbial relationships shift across the spectrum from pathogenesis to symbiosis, highlighting conditional pathogenicity and the shared molecular features of mutualists and pathogens. To date, there has been limited integration of examples of conditional pathogenicity with aquatic experimental models. Finally, we discuss host determinants of infection outcome and pathogen strategies that manipulate or evade host defenses, highlighting that route of infection is a potential determinant of host outcome. Aquatic models reveal that pathogenesis is not simply a binary state based on properties of a microbe or a host, but the result of dynamic interactions.

## 2. Defining Aquatic Models

Host–pathogen interactions involve the interplay of pathogenic factors, host responses, and environmental variables. As such, multiple invertebrate and vertebrate models have been developed to better model the interplay between all three. Invertebrate models offer the advantages of ease of use and low cost, while vertebrate models are often used to bridge the gap between the tractability of invertebrates and the complexity of mammalian hosts, increasing translational potential.

Aquatic invertebrate models include bivalves (e.g., oysters), anthozoa (e.g., sea anemones), cephalopods (e.g., squid), and crustaceans (e.g., brine shrimp). Although many of these models have some transcriptomic resources, sea anemone and brine shrimp have more extensive transcriptomic resources [4,5,6,7,8]. As such, it is perhaps unsurprising that sea anemones now also serve as models for regeneration and evolution in addition to host–pathogen interactions [9]. The two most used sea anemones to model host–pathogen interactions are *Aiptasia pallida* and *Nematostella vectensis*. *A. pallida* can withstand at least a 5 °C temperature change, which is the predicted seawater surface temperature change in the next twenty years [4]. The capability to withstand the increase in water temperature provides a model to study how microbes respond to environmental changes and how pathogens that thrive in these conditions may pose a concern for human infection. Comparing the different growth rates of the two species, researchers have also found that the presence of symbionts can slow the shrinkage rate of *Aiptasia*, highlighting the advantage of using these two species for comparative host–pathogen interactions [10]. *A. pallida* is widely used to study the response to coral-killing bacteria, namely *Vibrio* species, which have increased presence due to the rise in seawater temperature [4]. In contrast, *N. vectensis* has general resilience to pathogens, most likely due to the genome encoding one toll-like receptor (TLR) [11]. Indeed, exposure to heat-killed *Vibrio coralliilyticus* activates NF-κB signal transduction, providing a simple model to study bacterial immunity at a molecular level [11,12]. Building on this, the effect three different bacterial species (*Bacillus velezensis*, *Vibrio diabolicus*, and *Pandanus spiralis*) had on *N. vectensis* was recently characterized [13]. Although some core gene expression changes were observed for all treatments, large transcriptional responses were observed for *B. velezensis* and *P. spiralis* but not *V. diabolicus*. Further, these changes were dependent on the host microbiome, highlighting the complexity of host–pathogen interactions and the utility of using a simpler model organism to tease out these important factors [13].

Like *A. pallida*, *Artemia* brine shrimp species are found in salt water and can tolerate any level of salinity between 2.5% and 25% [6]. This is beneficial in modeling host–pathogen interactions that evolve as both host defenses and pathogens adapt across salinity concentrations [14,15]. A recent study using artificial sea salt contaminated with microplastics determined the effects *Vibrio harveyi* had on brine shrimp development and enzymatic activity [16]. This work highlights the utility of using an invertebrate model for complex interactions between a host, pathogen, and the environment. Brine shrimp offer additional advantages as well. For example, brine shrimp can switch between reproducing ovoviviparously or oviparously [17,18]. This permits the study of how pathogens may be transmitted from maternal infection, as well as determines how microbiome/pathogen transmission compares across live-born versus cyst-derived offspring [19]. The widespread use of brine shrimp as larval feed has led to the development of germ-free models, to prevent transmission of pathogens to hatchery-raised fish. Germ-free raising of brine shrimp permits purposeful flora colonization to determine how native flora can prevent or exacerbate pathogen infectivity [16,20,21]. Some of these advantages provide the opportunity to expand past host–pathogen interaction studies in brine shrimp, with research branching into toxicity screening of antimicrobials, metals, and bioactive compounds [22,23,24,25].

Although sea anemones and brine shrimp provide genetically tractable models to study the molecular drivers of host–pathogen interactions, they are less translationally relevant to humans. The remaining aquatic invertebrates bridge the gap to better model foodborne illness. These animals act as natural reserves for *Vibrio* species, algal toxins, and parasitic worms [26,27,28,29,30]. Established models of host–pathogen interactions include the Pacific oyster, the Mediterranean mussel, and the spineless cuttlefish [31,32,33]. Pacific oysters and Mediterranean mussels are major farmed aquaculture species. The Pacific oyster, *Crassostrea gigas*, is at high risk of exposure to pathogenic microbes through air and seawater with mass mortality events occurring in warmer seasons. This provides the opportunity to study how host immune systems change with environmental temperature changes [34,35,36,37,38,39]. In contrast, *Mytilus galloprovincialis*, the Mediterranean mussel, is not affected by mortality events and is more resistant to infection through the capability to trap and kill invading microbial pathogens [40]. A recent comparative study of the microbiota profiles of co-cultured *C. gigas* and *M. galloprovincialis* highlights differences in two species that may explain part of the difference in susceptibility [41]. *Vibrio* species made up a larger amount of the *C. gigas* microbiota compared to *M. galloprovincialis* which was dominated by *Pseudoalteromonas* species. This is one example that suggests host physiology can play an important role in suppressing or promoting colonization by pathogenic *Vibrio*.

The spineless cuttlefish, *Sepiella japonica*, is the most susceptible of the foodborne illness models to waterborne pathogens as intraspecific reproduction has caused a loss of genetic diversity and a reduction in pathogen resistance [32,42]. Additionally, the predatorial behavior of the cuttlefish leads to damaged skin, increasing its susceptibility to *Vibrio* infection [43]. Recent work has pointed to *V. harveyi* disrupting neuroendocrine-immune signaling through upregulation of the neuropeptide (FMRFamide) [44]. Identification of this peptide has opened the door to additional studies in *S. japonica*, including cloning and characterization of GABA receptors [45] and identification of additional neuropeptides [42,46,47]. The variation in susceptibility to pathogens between the established models for foodborne illnesses provides multiple platforms for comparative studies to determine virulence mechanisms.

Aquatic vertebrate models include amphibians (e.g., frogs and axolotls), fishes (e.g., zebrafish), reptiles (e.g., sea turtles), and mammals (e.g., dolphins). Although studying host–microbe interactions in marine reptiles and mammals is challenging, both case studies and longitudinal studies have identified pathogens that are known to infect these aquatic species and humans [48,49]. For example, sea otters are reservoirs for *Vibrio* species that have variable virulence factors [50]. For the remainder of this review, vertebrate models refer to species that can be manipulated in a laboratory setting.

Vertebrate models have physiological and genetic complexity similar to humans, offering a “sweet spot” between scalability of smaller species but with organ systems that invertebrates cannot recapitulate. For example, like *A. pallida* and brine shrimp, *Xenopus laevis* are also able to adjust to hypertonic environments, allowing for the study of salinity adapting pathogens [51]. Amphibians and teleost fish have transparent larvae and the capacity to be genetically manipulated. This ability has made frogs, specifically the *Xenopus* species, commonly used models for developmental biology [52,53,54].

Both the innate and adaptive immune systems in *Xenopus* have conserved similarity to humans [55]. Unlike other aquatic species, the adaptive arm is more extensively characterized than the innate arm [56]. One conserved cell type of the adaptive immune system is T cells; not only are T cells conserved, the unique subset of innate-like T (iT) cells are conserved as well. Interestingly, these iT cell subsets are predominant in tadpoles whereas metamorphosis leads to a change in T cell populations [57]. Therefore, *Xenopus* tadpoles provide the opportunity to study the cellular immune response to pathogens in the presence and absence of traditional T cell populations [57]. Although the adaptive immune system is more characterized than the innate system in *Xenopus*, there are specific instances where the innate branch has been the focus of studies, for example with the ranavirus. The ranavirus pathogen, which infects amphibians, reptiles, and fish, has been shown to evade the frog’s immune system [58,59,60,61]. Further, scientists found that the virus can hide dormant in macrophages, leading to immune evasion [60,62]. This serves as a new model to study other pathogens such as hepatitis C, which can hide dormant in liver macrophages [63]. In addition to serving as an immune system model, *Xenopus* offer the additional advantage of producing antimicrobial peptides [64,65,66]. Frog skin-derived antimicrobial peptides have been shown to protect against *Pseudomonas aeruginosa* infections, providing a new model for drug discovery [67].

Like *Xenopus*, teleost fish also provide a powerful platform to study innate and adaptive immune responses in an optically transparent animal. One teleost that is growing rapidly for laboratory use is medaka. Medaka species have evolved in both freshwater and saltwater environments, allowing for comparisons of host immune development and response in different salinities to the same pathogen [68,69,70]. For example, the gut microbiome is different between *Oryzias minutillus* (freshwater) and *Oryzias javanicus* (brackish water), leading to differences in immune system development and resistance to pathogens [71,72]. Medaka are also well suited for studying host–pathogen interactions under climate stress. The Japanese medaka is capable of a reversible, rapid plasticity response to heat waves and has demonstrated multigenerational adaptation to increased water temperatures [73,74,75]. This capability is important to study how temperature changes the immune response in addition to the study of different pathogens that have increased virulence in different temperatures. In the context of *Vibrio*, this capability is particularly powerful as virulence increases with higher water temperatures [76]. Medaka also reduce the complexity and difficulty in studying host genetics in response to a pathogen as this model has a smaller genome size compared to other teleost fish and *Xenopus* [77]. Overall, the medaka provides a powerful model because it links infection biology with immunology, microbiome biology, environmental salinity, and climate adaptation in a genetically and experimentally accessible vertebrate model.

To date, the flagship teleost model for studying host–pathogen interactions is the zebrafish. Zebrafish offer additional advantages over medaka such as earlier hatching, around 48–72 h post-fertilization. This early developmental window and transparent embryos allow non-invasive, high-resolution live imaging of infection dynamics. For example, GFP-labelled *Vibrio anguillarum* was used to determine a route of invasion in water containing increasing amounts of bacteria [78]. In this work, *V. anguillarum* was detected in the gastrointestinal tract first, even though previous studies suggested involvement of skin and gills [79,80]. Imaging of infection showed that the aquatic pathogen *Pseudoloma neurophilia* also causes granuloma formation [81,82,83]. These granulomas develop in the brain, leading to neurological deficits in zebrafish [84]. This outcome mirrors the granulomatous meningoencephalitis seen in mammals infected with *Encephalitozoon cuniculi*, providing a foundation for the use of zebrafish as a comparative model to mammals to study host susceptibility and routes of infection [85].

Further, the external development of zebrafish embryos provides the opportunity to study embryonic, prenatal-like, or perinatal infection in a vertebrate outside the maternal body, avoiding effects of placental immune responses [86,87,88]. Just as brine shrimp have the capability of being raised germ-free, zebrafish can be as well, allowing for determination of the host response to a specific pathogen and the ability to study gut microbiomes [89]. In a recent study comparing germ-free and conventional zebrafish microbiota, it was demonstrated that a community of bacterial strains protected the embryos from infection with *Flavobacterium columnare* [90]. Further, this study found protection did not rely on modulation of the immune system, highlighting the complexity of aquatic interactions which occur in the presence of multiple species. This is in contrast to work showing that zebrafish microbiota have many effects on the immune system including neutrophil abundance and expression of pro-inflammatory genes [91]. Continued research on the zebrafish microbiota opens up the possibility of investigating the role of probiotics as a protective mechanism for both fisheries and research laboratories [92].

Similar to *Xenopus*, zebrafish have well characterized immune systems with temporally separated innate and adaptive arms [93,94]. Additionally, the genetic tractability of zebrafish [95] has led the research community to develop a large number of transgenic and knockout lines to determine the host cellular response to infection [96,97]. Transgenic lines labeling immune cells have been used in unison with live imaging to study the progression of infection over time and characterize the immune response to infection [98,99]. Using a transgenic line that labels blood vessels, *Mycobacterium abscessus* was observed to absorbed from the bloodstream into granulomas previously established by *Mycobacterium marinum* [100]. Similarly, knockdown and knockout lines permit the investigation of infection in the presence or absence of different populations of innate immune cells [101] and signaling pathways. With all these advantages, it is perhaps unsurprising that zebrafish are used to study a spectrum of pathogens from aquatic-specific (e.g., *Vibrio*) to clinically relevant (e.g., *Mycobacterium*) [102,103,104,105,106,107,108,109,110,111].

Together, these features make zebrafish one of the most versatile aquatic models to host–pathogen interactions. Importantly, zebrafish can be used to study both natural aquatic pathogens and clinically relevant microbes, making them valuable for comparative infection biology, aquaculture health, and human disease modeling. As a result, zebrafish provide a bridge between ecologically relevant aquatic host–pathogen systems and mechanistic vertebrate immunology.

The diversity of aquatic models provides a unique opportunity to study the ever-changing landscape of host–pathogen interactions. Together, aquatic models provide complementary ways to manipulate the three determinants of interaction outcome. Model selection can determine which components of the host–microbe-environment interaction can be isolated experimentally.

## 3. Microbial Plasticity—From Pathogenesis to Symbiosis

To broadly describe bacterial behaviors, species are often categorized based on phenotypes or lifestyles. Pathogen and symbiont are terms often used when describing the interactions between a microbe and a host organism. A pathogen is defined as a microbe capable of causing disease or measurable harm in a host [112]. In contrast, a symbiont is any organism living in persistent and close physical proximity with another species [113]. Symbiotic relationships include three categories: (1) parasitism, where one partner benefits at the expense of the other; (2) commensalism, where one partner benefits without affecting the other; (3) mutualism, where both partners benefit from each other. A mutualist will typically supply its host with some functional advantage, such as protection and nutrition, in exchange for shelter and reliable access to resources it could not otherwise obtain [114]. Though these categories, symbiont and pathogen, are often presented as fixed and separate ways of life, the molecular mechanisms underlying them overlap far more than the labels suggest [113].

The *Vibrio* genus offers a clear illustration of this full range, since its member species span nearly the entire spectrum from pathogen to mutualist (Table 1). *Vibrio* is a genus of Gram-negative, comma-shaped rod bacteria distributed throughout marine and freshwater environments worldwide. Members of this genus are facultative anaerobes with a single polar flagellum that drives motility. More than 100 *Vibrio* species have been identified and described [115]. These species occupy a wide range of lifestyles, with some living freely in open water and the surrounding water column and others forming parasitic or pathogenic relationships with fish, invertebrates, or humans [115]. Still other vibrios establish long-term mutualistic relationships with their host [115]. *Vibrio coralliilyticus* is a coral pathogen also found in the sea anemones *A. pallida* [116] and *Nematostella vectensis* [11]. *Vibrio harveyi*, *Vibrio splendidus*, and *Vibrio alginolyticus* cause disease and mass mortality in the Pacific oyster *C. gigas* and related bivalves [117]. *Vibrio cholerae* is a major cause of cholera, while its relatives *Vibrio parahaemolyticus*, *Vibrio vulnificus*, and *Vibrio alginolyticus* are major causes of vibriosis [115,118].

While mutualism in *Vibrio* is rare, *Aliivibrio fischeri* (formerly *Vibrio fischeri*, hereafter *V. fischeri*) is a beneficial partner to the Hawaiian bobtail squid, *Euprymna scolopes*, and *Aliivibrio logei* (formerly *Vibrio logei*, hereafter *V. logei*) co-colonizes the light organ of squid in the genus *Sepiola* alongside *V. fischeri* [119]. The parenthetical genus name reflects a 2007 reclassification, in which *V. fischeri*, *V. logei*, and two related species were moved into a new genus, *Aliivibrio*, based on phylogenetic distance from other *Vibrio* species [120]. While *Vibrio* and *Aliivibrio* nomenclature are both in use, the squid symbiosis literature continues to utilize *V. fischeri*. For this review, we will use the *V. fischeri* nomenclature to align with the symbiosis literature while recognizing the current taxonomy of *Aliivibrio*.

Most mechanistic work on the *Vibrio* genus’s role in disease concentrates on *V. cholerae*, *V. parahaemolyticus*, and *V. vulnificus*, since they cause the clearest human disease burden, while the deepest mechanistic work on symbiosis itself has concentrated on *V. fischeri*, whose partnership with the squid light organ remains the best-studied mutualism [121,122]. However, *Vibrio*’s presence across aquatic host systems indicates that its biology is broader than a narrow focus on human disease would suggest.

This duality where a single organism can act as both a pathogen and a beneficial partner extends beyond *Vibrio; Aeromonas* species display a similar functional dichotomy. *Aeromonas veronii* acts as a beneficial gut symbiont in the medicinal leech *Hirudo verbana*, where it assists in blood digestion and secretes antimicrobial compounds to protect the host [123]. However, *A. veronii* is also a well-documented pathogen of fish and humans, causing septicemia, wound infections, and gastroenteritis, while simultaneously functioning as a gut symbiont in zebrafish [124]. Other members of the genus are predominantly pathogenic. *Aeromonas hydrophila* causes motile *Aeromonas* septicemia in freshwater fish and can also cause gastroenteritis, wound infections, and septicemia in immunocompromised humans [125]. Similarly, *Aeromonas salmonicida* causes furunculosis in salmonid fish and remains one of the most economically damaging pathogens in global aquaculture [126]. All these examples demonstrate that the genus *Aeromonas* spans a continuum from mutualism to pathogenicity, with similar host-association mechanisms supporting markedly different outcomes depending on the species and host context.

### 3.1. Moving Beyond the Pathogen Versus Non-Pathogen Binary

**Figure 1:**
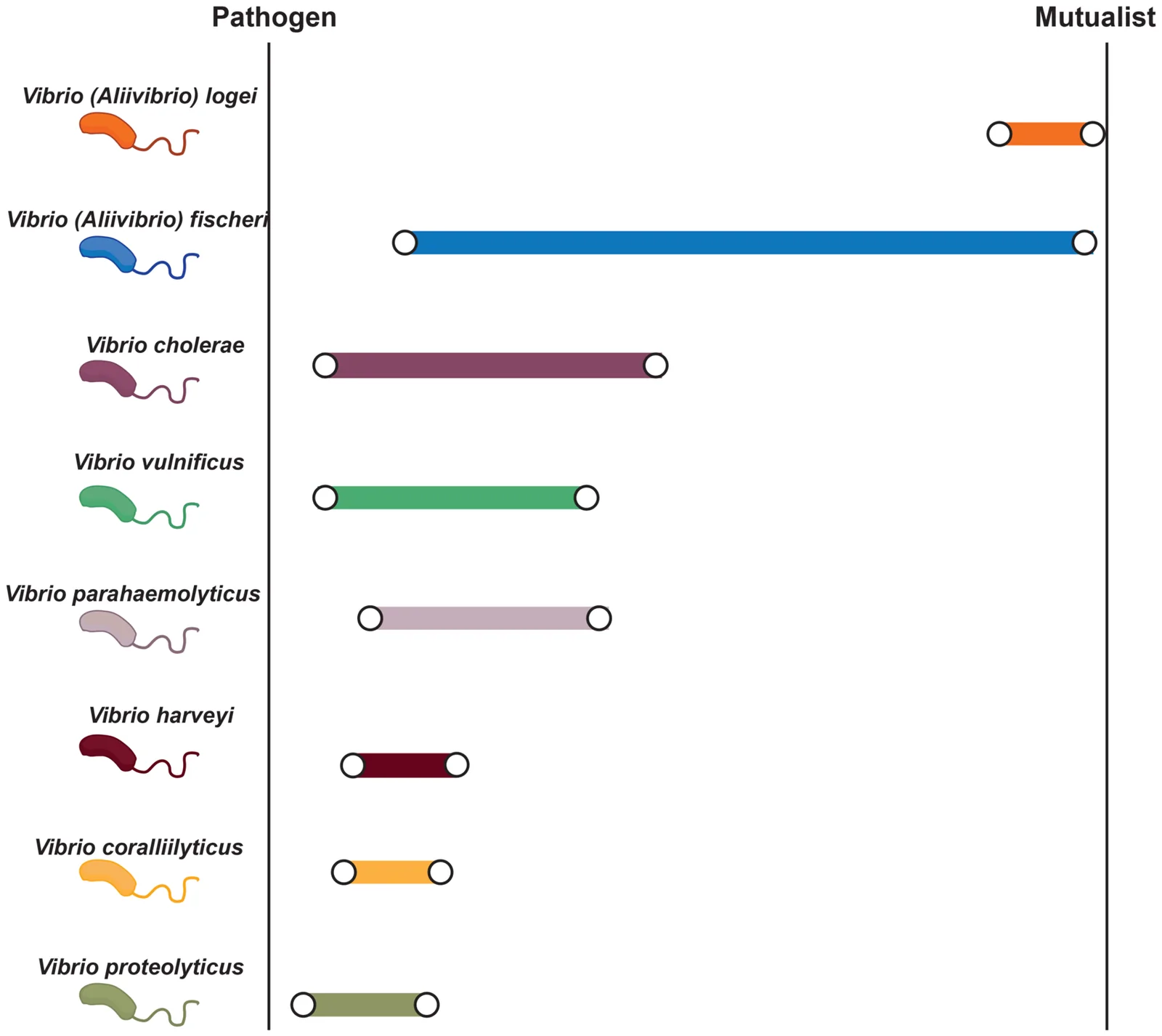
**Conceptualization of *Vibrio* species range from pathogens to mutualists.** Many *Vibrio* are not isolated to a single category on the pathogen vs. non-pathogen spectrum. Position of each baris based on the reviewed literature rather than representing a calculated value, formal metric, or quantitative disease severity score. Bar length reflects the extent of evidence-based variation for each species. Longer bars indicate substantial behavioral shifts across hosts or conditions, whereas shorter bars represent a narrower documented range rather than a fixed position. Schematic was made using Adobe Illustrator 2026.

Koch’s postulates, formulated in the 1880s, established the traditional framework for understanding microbial disease [127]. Under this framework, a microorganism either causes a specific disease or it does not. That capacity is treated as a fixed trait of the microbe itself. The framework was foundational to the germ theory of disease, and identified the causative agents of tuberculosis, cholera, and anthrax. As microbiology advanced with tools like PCR and in situ hybridization, the limits of the pathogen vs. non-pathogen binary view became apparent. Asymptomatic carriage and organisms that cannot be cultured on artificial media do not fit neatly into a framework built on strict yes-or-no causation [127]. A more recent reframing, called ecological Koch’s postulates, goes further by arguing that pathogenic effects cannot be understood apart from the surrounding microbiome and host ecology. The same organism can cause disease in one setting, colonize without symptoms in another, or do nothing at all, depending on the resident microbial community it enters [128] (Figure 1).

Virulence emerges from the specific interaction between a host and a microbe. Pathogenicity within a single species can vary enormously depending on which host and bacterial strain are involved [129]. *V. cholerae* illustrates this well at the strain level. Strains in the O1 and O139 serogroups predominately carry the CTX phage that encodes cholera toxin and the toxin-coregulated pilus needed to colonize the intestine [130]. The O1 and O139 strains are responsible for epidemic cholera, while most non-O1/non-O139 strains lack these toxins and generally cause milder or no disease in humans [131]. *Staphylococcus aureus* exemplifies how specific host interaction changes pathogenic outcomes. *S. aureus* colonizes the nose of a large fraction of healthy people without ever causing disease [132]. However, once it enters the bloodstream, *S. aureus* can cause an invasive infection and death [132]. What separates these outcomes are the host’s barriers and immune status, not a fixed property of the bacterium itself [132]. The damage-response framework formalizes this idea directly. It holds that the outcome of any host–microbe encounter, whether harmless colonization or lethal disease, is decided by the interaction between microbial factors and the host’s own immune response, not by the microbe alone. Virulence and pathogenicity, in other words, are not intrinsic microbial traits. They are outcomes of a specific host–microbe interaction, which is why the same organism can behave as a harmless commensal in one host and a lethal pathogen in another [129]. This host-dependence, evident at both the strain level and the individual level, is the foundation the rest of this review builds on.

### 3.2. Conditional Pathogenicity

Borrowed from game theory to describe infections as a payoff between host and microbe [133], the Focal Point Theory sorts host–microbe relationships into three categories based on how the payoff structure resolves. (1) Non-pathogenic microbes engage in a pure cooperative game, in which both host and microbe come out ahead. (2) Unconditional pathogens engage in a pure conflict game, where the interests of host and microbe are directly opposed no matter the situation. (3) Conditional pathogens sit in a dilemma game, where the same interaction can resolve toward cooperation or conflict depending on the specific conditions involved. These conditions can be host-related, such as immune status or organ health. They can also be environmental, such as temperature or nutrient availability.

Conditional pathogenicity is the clearest expression of host-dependent pathogenicity. A microbe’s capacity to cause disease depends on the host, the environment, or the immune conditions it encounters rather than on a fixed property it always expresses. This dependence usually traces back to two distinct mechanisms. One is which genes a microbe carries. The other is how those genes get regulated. Unconditional pathogens typically carry a core set of virulence genes and express them reliably whenever they meet a susceptible host. For conditional pathogens, sometimes only certain strains carry the relevant virulence genes, as in *V. cholerae*. Other times most strains carry the genes but keep expression gated behind specific environmental or host cues.

*V. vulnificus* shows how conditional pathogenicity can shift with the host’s own health. It rarely causes more than mild gastroenteritis in a healthy adult. The same bacterium can cause septicemia with a mortality rate exceeding 50% in a person with chronic liver disease, diabetes, or an iron overload condition such as hemochromatosis [134]. Impaired liver function leaves more free iron circulating for the bacterium to use. *V. parahaemolyticus* reaches a similar outcome through a different route. This route works through gene presence and absence at the strain level rather than host health. Only an estimated 0–6% of environmental isolates carry the *tdh* and *trh* genes responsible for the gastroenteritis this species is known for [135]. Strains lacking these genes are generally less virulent, though not uniformly harmless. Some non-toxigenic strains carry a separate toxin, zonula occludens toxin, that disrupts host cells independently [136].

This strain-specific logic of virulence is also observed in *Legionella pneumophila*, an aquatic bacterium found in natural biofilms and built water infrastructure [137]. The species exhibits pronounced intraspecific variation where clinical isolates display higher cytotoxicity than environmental isolates, and distinct environmental genotypes yield variable virulence in amoebic and macrophage models [138]. Moreover, *L. pneumophila* exemplifies the training ground hypothesis given that its long evolutionary pressure from freshwater amoebic hosts equipped the organism with intracellular survival strategies later adapted to infect human macrophages and cause Legionnaires’ disease [139].

*V. fischeri* illustrates this most dramatically of any species in Figure 1. Rather than sitting at one end of the range, it spans nearly the entire axis. Nearly every strain retains virulence-associated genes closely related to those in *V. cholerae*, *V. parahaemolyticus*, and *V. vulnificus*. These genes stay switched off under most conditions [121]. The clearest demonstration of this comes from the zebrafish–*V. fischeri* system. A dedicated secretion system, T6SS1, makes the bacterium lethal to both zebrafish embryos and brine shrimp. A separate system, T6SS2, instead supports cooperative behavior inside the squid light organ [140,141]. The species most associated with mutualism turns out to be one of the best-documented pathogens in the genus.

Two additional species (Figure 1) complicate this picture rather than resolve it. *V. coralliilyticus* and *V. harveyi* are both documented pathogens, but neither has been shown to always cause disease. Neither species has a milder or symbiotic state documented anywhere in the literature. Their position toward the pathogenic end of the spectrum reflects the evidence currently available about them. It is not a claim that their behavior is fixed the way an unconditional pathogen’s is. This uncertainty extends beyond these two species. The categories applied throughout this review, such as conditional, unconditional, or mutualistic, reflect currently available evidence for each species rather than definitive biological designations. For many *Vibrio* species, host specificity and the drivers of pathogenicity remain far less characterized than in model organisms like *V. cholerae*, *V. parahaemolyticus*, or *V. fischeri*. Consequently, a species positioned toward one extreme of the spectrum may genuinely lack documented exceptions, or it may simply reflect a lack of targeted research.

The host can also actively steer a microbe’s behavior. In a study using germ-*free Drosophila melanogaster* larvae colonized with the opportunistic bacterium *Serratia marcescens*, the fly larvae actively drove the bacterium to switch from a pathogenic to a commensal lifestyle. This switch reshaped its transcriptomic and metabolic profile in the process [142]. Antimicrobial peptides produced by the larvae were required to reproduce this switch. Single-cell sequencing showed the response was not uniform across the bacteria. Individual bacterial cells shifted their behavior to different degrees depending on the specific signals they received from the host. The outcome of an infection is not fixed the moment the bacterium is introduced. The host can shape bacterial behavior cell by cell.

### 3.3. Symbiosis as a Framework for Host–Microbe Interactions

Host-dependent outcomes shift the emphasis away from the microbe and onto the relationship the bacteria forms with a given host. Symbiosis offers one of the clearest existing frameworks for describing the host–microbe relationship, since it is derived from the host and microbe co-constructing a shared physiological state. A growing body of work presents symbiosis and pathogenesis less as opposite ends of a spectrum and more as different outcomes of the same underlying biology [113].

The *V. fischeri*–*E. scolopes* symbiosis is a model system that helped establish that a beneficial association could be studied down to the same molecular detail as pathogens [121,122]. *V. fischeri* is the exclusive bacterial symbiont of the Hawaiian bobtail squid and colonizes the squid’s light organ [121,122]. Colonization of the light organ unfolds in three distinct stages. These are initiation, colonization, and persistence. Juvenile squid hatch with no symbiont and must take up free-living *V. fischeri* from the surrounding seawater. The squid does not passively accept whatever bacteria it encounters. Instead, the squid actively selects for *V. fischeri* through host-bacterial specific signaling. Bacterial peptidoglycan first triggers the host to shed mucus that facilitates initial *V. fischeri* attachment and aggregation. The squid then uses antimicrobial peptides and reactive oxygen species to eliminate all bacteria except *V. fischeri* [121]. Once established in the light organ, *V. fischeri* gains a nutrient-rich environment. The squid in turn gains access to *V. fischeri*-produced bioluminescence, which it uses for counter-illumination camouflage.

Despite the *V. fischeri*–squid interaction resulting in a symbiotic relationship, *V. fischeri* uses behaviors for squid colonization that overlap with concepts of mutualism and pathogenesis. *V. fischeri* produces tracheal cytotoxin, the same toxin *Bordetella pertussis* uses to cause whooping cough by damaging and killing the ciliated cells lining the human respiratory tract [143]. In *V. fischeri*, this same toxin instead triggers beneficial morphological development in the squid’s light organ [121]. The *V. fischeri* genome also carries homologous genes to several *V. cholerae* toxin and stress-response genes, including *toxR*, *toxS*, *htpG*, and *rpoH9*. The *V. cholerae* virulence regulator TcpP is homologous to *V. fischeri* HbtR, but rather than activating virulence genes, HbtR helps *V. fischeri* switch between its symbiotic and free-living lifestyles, a repurposing of a virulence-regulatory system for a mutualistic function [144].

Although microbes are often labeled as pathogens or symbionts, these categories can obscure the context-dependent nature of host–microbe interactions. The genus *Vibrio* illustrates this complexity, with species ranging from human and aquatic pathogens to the mutualist *V. fischeri*. This relational view raises the following question: which host determinants shape whether a microbial encounter remains harmless, becomes beneficial, or progresses to disease?

## 4. Microbial Determinants of Pathogenicity

While the previous sections focused on the host’s role in shaping infection outcomes, the microbe contributes equally. As defined by the training ground hypothesis, pathogenicity is a byproduct, not the reason the virulence trait evolved. A microbe often evolves certain traits to survive predation by environmental protozoa [145]. Those same traits happen to confer pathogenic ability the moment the microbe meets an unfamiliar animal host [145]. As an example of the training ground hypothesis, in the wild *V. cholerae* and *V. harveyi* are regularly engulfed by protozoan predators [146]. These predators package bacteria into phagosomes and expose them to low pH, antimicrobial peptides, reactive oxygen species, and iron limitation [146]. The bacteria evolved specific traits to survive ingestion, including resistance to acid stress, antimicrobial peptides, and oxidative damage [146]. Immune cells in an animal host rely on similar killing mechanisms [146]. Therefore, tools evolved for escaping a predator in an aquatic system work similarly for escaping an animal host’s phagocytic immune cells.

### 4.1. Classic Virulence Factors

#### 4.1.1. Adhesins

Adhesion is usually the first step in establishing a *Vibrio* infection. A bacterium must attach to a host cell or tissue surface before it can damage that cell or establish a stable niche inside it. *V. parahaemolyticus* attaches itself to corals, fish, oysters, sponges, shrimp, and zooplankton in addition to human hosts [147]. Adhesion mechanisms vary across the genus. Some *Vibrio* species rely on dedicated surface adhesins, while others depend on flagella-driven motility that brings a bacterium into contact with host tissue [148].

*Vibrio* adhesion also leads to virulence traits including toxin delivery and immune invasion [149]. *V. parahaemolyticus*, *V. harveyi*, and *V. campbellii* use adhesins that bind fibronectin and fibrinogen on the host cell surface [147,150]. Deleting the *V. parahaemolyticus* adhesin *vpadF* sharply reduces the bacteria’s ability to attach to and damage epithelial cells, and virulence in a mouse model drops correspondingly [147]. Attachment is a prerequisite for the cytotoxicity and tissue damage that follows, so a mutation that blocks adhesion blocks virulence as a direct consequence on disease outcomes. Similarly, in *V. vulnificus*, adhesion mechanisms contribute to host cell and tissue invasion [149]. Together, these findings show that adhesion is more than a simple attachment step in *Vibrio* pathogenesis. It is a gateway trait that links environmental persistence, host colonization, and virulence.

#### 4.1.2. Intracellular Survival

Some *Vibrio* species invade the host cell they attach to, a process known as facultative intracellular survival [151]. This allows many *Vibrio* species to exploit an intracellular niche despite being predominately extracellular pathogens. *V. bathopelagicus*, an oyster pathogen, uses intracellular survival in the marine amoeba *Paramoeba atlantica* as a protective environment away from protozoan predators [152]. *V. parahaemolyticus* exhibits intracellular survival mediated by the Type III Secretion System (T3SS) [151]. To avoid detection, *V. parahaemolyticus* modifies its lipopolysaccharide, which protects the cell from host recognition [153]. Intracellular survival is an important but underexplored mechanism explaining *Vibrio* pathogenesis independent of aquatic host.

#### 4.1.3. Lipopolysaccharide

Lipopolysaccharide (LPS) coats the outer membrane of Gram-negative bacteria. LPS is one of the most immediately recognizable signals a host innate immune system detects [154]. Predominately recognized through TLR4 (Toll-like receptor 4), a host cell-surface receptor triggers inflammation upon binding bacterial LPS [154]. Several *Vibrio* species modify their LPS depending on the environment. *V. parahaemolyticus* produces a heavily acylated form of LPS as a free-living bacterium but shifts to a less-acylated form once inside a host cell, one that triggers a noticeably weaker TLR4 response [153]. Deleting the acyltransferase responsible for this transition, lpxM, alters the bacterium’s virulence. *V. fischeri* modifies its LPS in a comparable way upon encountering the mildly acidic pH of squid mucus [155]. *V. fischeri* uses the ethanolamine transferase eptA to modify the lipid A portion of its LPS, increasing resistance to host antimicrobial peptides and promoting successful colonization of the light organ [155]. Across the *Vibrio* genus, LPS is not a fixed structural feature. Instead, LPS is a dial the bacteria adjust depending on the environment. Perhaps adjusting LPS allows *Vibrio* species to tune their surface chemistries to different host environments, reducing immune detection while improving survival during colonization. However, this idea also raises important questions about which cues control LPS remodeling and whether the same LPS states that promote survival in marine hosts also enhance immune evasion during human infection.

#### 4.1.4. Immune Evasion

Bacteria use several distinct strategies to avoid detection or destruction by a host immune system. The LPS and intracellular-survival strategies described above are two examples where a bacterium either hides or alters its outer membrane to avoid host recognition and evade an immune response. Capsule production is third mechanism for evading the host immune system. *V. vulnificus* and *V. alginolyticus* rely on a capsular polysaccharide, a thick outer coating that blocks host complement proteins from tagging the bacterium for destruction and makes it physically harder for immune cells to engulf [156,157]. This same capsule creates a vulnerability elsewhere. A specific bacteriophage, HH109, uses the capsular polysaccharide of V. alginolyticus as its point of attachment, allowing it to infect and kill the bacterium [158]. Immune evasion is not cost-free. *V. vulnificus* strains that lose their capsule, whether through mutation or through a natural switch some strains make between encapsulated and unencapsulated states, become dramatically easier for the host immune system to clear [156]. Capsule formation in *V. cholerae* and *V. parahaemolyticus* is strain-dependent. Lastly, a pathogen can avoid phagocytosis by innate immune cells. For example, *V. vulnificus* can resist phagocytosis without having to produce extracellular products to disrupt macrophages [159]. The ability of a bacterium to evade the host immune system is often important for establishing an infection. Immune evasion mechanisms can provide the capacity for disease when environmental conditions, host susceptibility, and bacterial physiology align to favor host invasion.

#### 4.1.5. Iron Scavenging

Iron is abundant in the environment but rarely available in a chemical form bacteria can use directly, and free iron floating unbound in tissue is also toxic, since it drives damaging oxidative chemistry [160]. Host blood keeps iron tightly bound to proteins to starve out invading bacteria and to avoid iron toxicity. *Vibrio* species scavenge host iron through two predominate mechanisms [161]. Most species produce siderophores—small, secreted molecules that bind iron and transport it back to the bacteria. Each species produces its own siderophore, with vibrioferrin, anguibactin, and vulnibactin being produced by *V. parahaemolyticus*, *V. anguillarum*, and *V. vulnificus*, respectively [162]. Several *Vibrio* species also acquire iron directly from host heme and hemoglobin by secreting exotoxins to release the heme, bypassing the siderophore step entirely [162]. The utilization of both siderophores and direct heme uptake allows *Vibrio* species to effectively compete for iron and overcome this growth-limiting factor.

#### 4.1.6. Toxins

Toxins allow a pathogen to damage host tissue, disable an immune response, or acquire nutrients without needing direct physical contact with every cell the toxin affects [163]. Cholera toxin, the best-known toxin in the *Vibrio* genus, is a classic AB5 toxin that impacts host signaling proteins to drive a dramatic increase in cAMP production [164]. Increased cAMP pulls ions and water out of the cells lining the host’s gut and results in cholera’s characteristic watery diarrhea that facilitates *V. cholerae* survival through nutrient acquisition, competitor clearing, and enhanced transmission [165,166].

Other *Vibrio* species rely on different toxins, such as the *V. vulnificus* hemolysin VvhA that is a pore-forming cholesterol-dependent cytolysin that causes necrosis, apoptosis, and pyroptosis across a range of host cell types [167]. The related MARTX toxin, encoded by *rtxA*, damages host cells through a distinct mechanism [168]. In coral and shellfish systems, toxins take a different form again. An extracellular metalloprotease secreted by *V. tubiashii* and related coral pathogens is a major virulence factor against larval Pacific oysters [169]. Across the genus, the specific toxin and its target vary by species and by host. The underlying strategy does not vary. Secreting a molecule that damages the host directly is a tactic shared broadly across *Vibrio* species, and it benefits the bacterium in the same basic ways every time, namely by clearing space and competitors, releasing nutrients otherwise locked inside host tissue, and improving the odds of onward transmission to a new host. Importantly, toxin production and secretion are often tightly regulated and induced only under specific environmental, host-associated, or competitive conditions [170]. Therefore, the presence of a toxin virulence factor does not necessarily confer pathogenicity, but rather, disease outcomes depend on when and where the toxins are expressed.

#### 4.1.7. Secretion Systems

While toxins damage a host directly on contact, many bacteria also rely on dedicated machinery built to directly deliver toxins or effector proteins into a host cell. The T3SS works like a molecular syringe to inject bacterial effector proteins into the host and hijack the cell’s internal signaling systems [171]. *V. parahaemolyticus* carries two separate T3SS systems, with T3SS1 causing cytotoxicity and killing host cells outright while T3SS2 drives enterotoxicity [172]. The predominate secretion system used during a given infection depends on both the bacterial strain background and host context. This mirrors the conditional logic observed in toxin deployment rather than *V. parahaemolyticus* relying on a single, fixed pathogenic mechanism.

**Figure 2:**
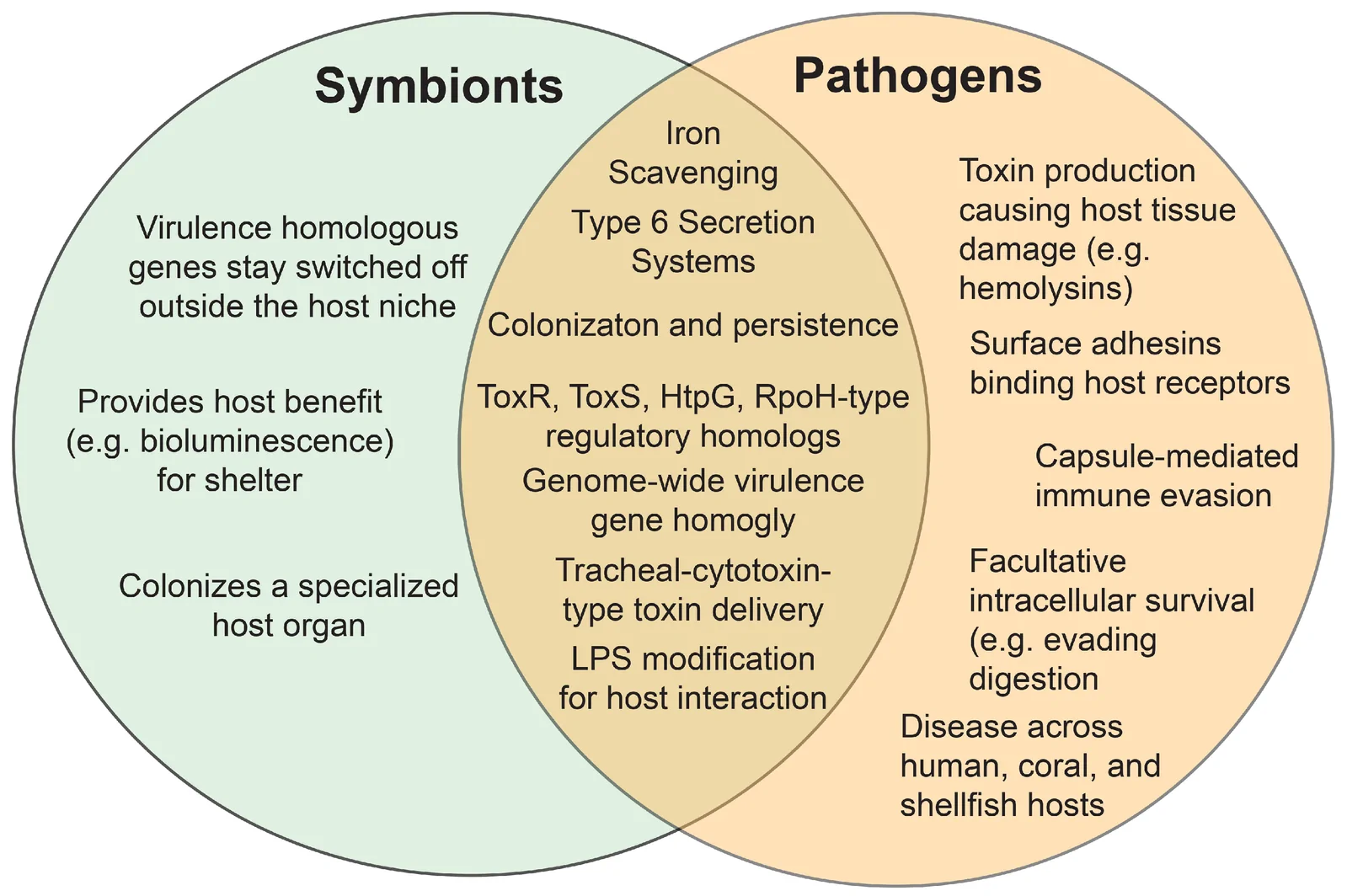
**Shared molecular toolkit between symbiotic and pathogenic *Vibrio* species.** The overlapping zone lists mechanisms documented in both categories.

Rather than injecting toxins directly into a eukaryotic host cell [173], the Type VI Secretion System (T6SS) first identified in *V. cholerae* [173] often functions as a contact-dependent weapon for eliminating competing bacteria [141,173], although some species use T6SSs to attack host cells or predatory amoeba [173]. *V. coralliilyticus* also encodes two T6SS systems, one that mediates interbacterial competition and a second distinct system that targets the coral or shellfish host directly and contributes to larval mortality [174]. Like *V. coralliilyticus*, *V. fischeri* also encodes one T6SS for interbacterial competition (T6SS2) and the other is proposed to be used against eukaryotic cells (T6SS1) [173,175]. *V. proteolyticus* was first identified as part of a bacterial consortium that causes yellow band disease in reef-building corals, alongside *V. harveyi*, *V. alginolyticus*, and *V. rotiferianus* [176]. *V. proteolyticus* has also been documented as a pathogen of brine shrimp by disrupting gut epithelial cells and entering the body cavity of infected nauplii [177]. Infection of corals and brine shrimp involves the T6SS. *V. proteolyticus* delivers toxin effectors directly into cells, with deletion of the effector genes sharply reducing shrimp mortality [178]. The fact that the secretion system used to cause coral disease also kills a crustacean host indicates that *Vibrio* did not evolve a new toxin-delivery system for each new type of host. An outstanding question is whether host-targeting systems evolved repeatedly from antibacterial systems or whether some systems were specialized early for eukaryotic interactions. Overall, the presence of multiple secretion systems in many *Vibrio* species that are involved in distinct processes (i.e., bacteria competition and host interaction) indicates that this virulence factor is context-dependent. The utilization of specific secretion systems is dependent on the environment and host interactions. These parallels, T6SS-mediated competition and host-targeting, LPS remodeling, iron acquisition, and shared regulatory homologs illustrate that pathogen and symbionts frequency draw from the same underlying molecular toolkit (Figure 2).

### 4.2. Context-Dependent Virulence

Classic virulence factors are often energy intensive and not switched on all the time [179]. Virulence gene expression is instead governed by specific environmental and host-derived cues [179]. This can result in the same bacteria behaving very differently depending on the conditions they encounter. This is the same regulatory gating discussed in Section 3 and refers to genes that are present in the genome but only expressed under a narrow set of circumstances.

For example, *V. cholerae* expression of both cholera toxin and the toxin-coregulated pilus, a surface appendage required for the bacterium to colonize the intestine, is controlled by a single regulatory cascade known as the ToxR regulon. This cascade integrates environmental signals including pH, osmolarity, and temperature, translating them into a coordinated increase in toxin and pilus production once the bacterium reaches the specific conditions found inside the small intestine [180]. The surrounding gut microbiome adds a further layer to this system, shaping *V. cholerae* virulence gene regulation alongside these host and environmental signals [170].

The *V. fischeri* T6SS2 also responds to specific environmental signals. It is activated by a combination of high viscosity and host-like pH, conditions found both in the mucus that coats the light organ during initial colonization and in the crypt spaces deep inside the organ where established symbionts compete for space [140,175]. This role is not interchangeable with T6SS1, where the environmental trigger has yet to be determined. Overall, across *Vibrio* species, virulence is often environmentally gated rather than constantly switched on, allowing the bacterium to control when specific virulence traits are utilized.

Altogether *Vibrio* virulence is best understood as context-dependent use of a shared molecular toolkit rather than as a fixed pathogen-specific program. Adhesion, intracellular survival, LPS remodeling, capsule production, iron scavenging, toxin secretion, and T3SS or T6SS activity can each promote infection, but these same traits also support environmental persistence, microbial competition, protozoan resistance, and colonization of diverse marine hosts. The central question is which ecological or host-derived cues activate these tools. Resolving this question will be essential for understanding why closely related *Vibrio* species can behave as pathogens or symbionts depending on the host and environment.

## 5. Host Determinants of Infection Outcomes

A combination of physiological, genetic, and environmental factors govern an organism’s susceptibility or resistance to a pathogen. Because aquatic models live in microbe-rich environments, both invertebrates and vertebrates evolved physical and physiological barriers and immune systems to protect themselves from infection. These models are powerful platforms to dissect the mechanisms that prevent pathogen entry, activate immune cells, and recover from tissue damage.

### 5.1. Barriers as a First Line of Defense

Aquatic models developed physical barriers to block pathogens from entering internal tissues. These include mucus, cell junctions, and scales. Mucus first evolved in marine invertebrates and is evolutionarily conserved through humans, highlighting its importance to defending all hosts from microbial invasion [181,182,183]. As such, some species have evolved mechanisms to breach the mucus layer. For example, *Vibrio* species are attracted to mucus and can use mucin, a component of mucus, as an energy source [184,185,186,187]. An ongoing active area of research is understanding when mucus acts as protection and when it creates a niche that inadvertently helps pathogens persist. Other methods of physical defense diverge between aquatic models. Aquatic invertebrates typically lack scales, instead using shells or exoskeletons as a defense, and form septate junctions to control water and solute movement between cells [188]. Whereas fish and amphibians have scales and have tight junctions which utilize claudin and occludin proteins to provide continuous sealing around cells [189]. Unfortunately, tight junctions are not fool-proof as breakdown due to continued inflammation results in a “leaky” barrier, allowing bacteria through. Further, pathogens are constantly evolving to overcome tight junctions. Some pathogens, like the *Mycobacterium* species, can directly bind to tight junctions, causing reorganizing and eventual entry into the brain tissue [106,190,191]. Others, such as *V. parahaemolyticus*, can release toxins and proteases to break down tight junction proteins to gain entry [136,192]. Together, physical barriers provide a critical first line of defense to the host; however, understanding whether barrier defects drive a host’s susceptibility to infection, how microbes strengthen or weaken physical barriers, and how barriers can be strengthened without causing harm to the host are still unanswered questions in the field.

Since physical barriers can fail, hosts have developed physiological barriers as a second line of defense. Physiological barriers are dependent on specific tissue types. Perhaps the most well-known physiological barrier is the gut. The gut acts as a barrier to infection through gastric or stomach acid. Gastric acid creates a highly acidic environment in the stomach; with a pH less than 3 leading to death of the majority of bacterial pathogens within minutes [193]. For example, *V. cholerae*, *Citrobacter rodentium*, and *Salmonella enterica* are all foodborne bacterial pathogens that are sensitive to gastric acid and are often killed prior to infection [193,194,195]. However, these pathogens can bypass the gastric acid defense when an organism’s stomach is less acidic, there is an increased bacterial load, or if these species develop an acid adaptation response [196]. In addition, the gut microbiota provide protection from pathogens as well. The gut microbiota serves to exclude pathogens metabolically by using local resources and producing antimicrobial molecules such as toxins and pH lowering compounds [197]. In some species, like zebrafish and tilapia, the gut microbiota also generates community-level resistance to certain pathogens (e.g., *Flavobacterium columnare*) [90]. Microbiota as a defense is not specific to vertebrates, as the Pacific white shrimp can develop resistance against *Vibrio* infections through colonization of *Shewanella algae* [198]. Interestingly, *S. algae* is considered an emerging human pathogen [199]; highlighting how a single bacteria can be part of a two-sides of the same coin: providing protection in once species and causing infections in another.

The gut microbiota does not provide resistance to all pathogens, for example *V. cholerae* is capable of displacing host gut microbiota to produce an infection, similar to cholera infection in humans [166]. Along with the gut microbiota, the zebrafish epithelium has gut-associated specialized enterocytes to trap bacteria in the intestine preventing bacterial translocation and infection [200]. In addition to the gut, vertebrates also have the blood–brain barrier (BBB). This selectively permeable barrier separates the brain and cerebral spinal fluid (CSF) from circulation. This filter for the brain is created around capillaries in three main layers with endothelial cells immediately against the capillaries then a basement membrane and pericytes and astrocytes as the last layer [201]. In zebrafish the BBB is capable of preventing exchange of large molecules by 3 days post-fertilization (dpf); allowing for studying of BBB protection against pathogens or failure during development [111]. This barrier prevents microorganisms from entering the brain; however, there are some pathogens that are capable of evading this barrier. Pathogens are capable of bypassing this physiological barrier through the Trojan horse mechanism [111], through paracellular migration when BBB integrity is weakened [111], or, like *Naegleria fowleri*, by entering the brain through the nasal cavity [202]. Just as physical barriers create a critical passive defense to fighting off pathogens, these physiological barriers provide a secondary passive defense prior to the involvement of the active defense system, the innate immune system.

### 5.2. Innate Immune System as Major Advantage

Given that all marine organisms are constantly exposed to pathogens, it is perhaps unsurprising that both invertebrates and vertebrates rely on immunity as their active defense against pathogens. Invertebrates have only an innate immune system whereas vertebrates develop adaptive immunity after development. This temporal resolution provides a unique opportunity to tease apart the cellular and molecular mechanisms that occur during initial infection, chronic exposure, and tissue repair.

### 5.3. Specialized Cells

Brine shrimp and bivalves have a single specialized cell type that is responsible for host immune defense. These cells are hemocytes, which recognize and engulf pathogens, produce cytotoxic molecules and antimicrobial peptides, and secrete inflammatory cytokines similar to the vertebrate immune system. Similar to innate immune cells in vertebrates, these cells also play a role in wound healing and detoxification [201,203,204].

**Figure 3:**
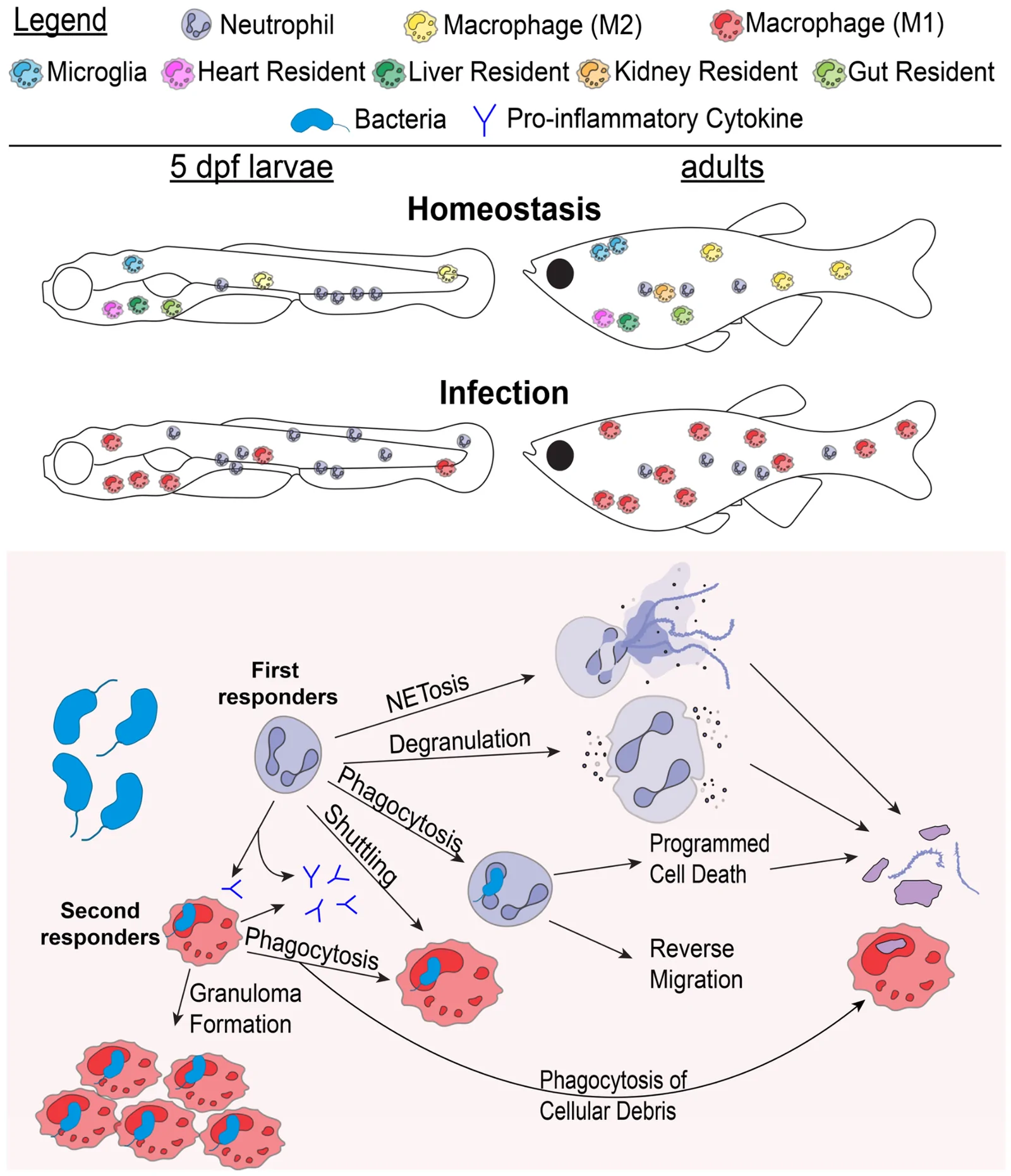
**Innate immune response to bacterial infection in zebrafish.** (**Top**): Locations of tissue-resident and systemic innate immune cells during homeostasis and infection in larvae and adult zebrafish. Resident macrophages are depicted are the brain, heart, liver, kidney and gut. (**Bottom**): Schematic of neutrophil and macrophage function in response to pathogens. Both neutrophils and macrophages produce inflammatory cytokines which recruit additional macrophages to clear the infection. Upon clearance, macrophages return to circulation, while neutrophils either return to circulation or go through programmed cell death. Schematics made with Adobe Illustrator.

Aquatic vertebrates are more like mammals who have many specialized cells (Figure 3). During immune responses these specialized cells are responsible for pro-inflammatory signaling in response to injury or infection, clearance of debris and pathogens, ending the inflammatory response after clearance, and the generation of memory signals to respond to future insults with increased efficiency [175,205,206,207,208]. The specialized cells seen in aquatic vertebrates and mammals are split into two branches of the immune system, the adaptive and innate systems [209]. The innate immune system cells are responsible for the production of pro-inflammatory signals, clearance of pathogens and debris and reducing inflammation upon clearance [205,210]. The cells of the adaptive system allows for antibodies to be generated for the pathogen and for prevention of future infection through immune cell memory and suppresses pro-inflammatory signals preventing excess damage [206]. For this section, we will primarily focus on zebrafish as they are the most widely used aquatic vertebrate model for studying host–pathogen interactions.

#### 5.3.1. Neutrophils

Of the specialized cells in the innate immune system, the first responders to injury and infection are neutrophils. Neutrophils migrate to the infection site to eliminate bacteria and signal for additional immune cells to clear the infection [211]. Neutrophil migration is guided by host-derived chemokines [212,213], bacterial products [214,215], and inflammatory lipid mediators [216], allowing neutrophils to move rapidly from the bloodstream into infected tissue. Once neutrophils arrive at the infection site or come in contact with a pathogen, they have multiple ways to respond, as depicted in Figure 3. Neutrophils can internalize the pathogen through phagocytosis, creating a phagosome containing the bacteria. To kill pathogens in the phagosome, neutrophils deliver antimicrobial granules to the phagosome, and the NADPH oxidase complex within the phagosome generates ROS [98,217]. These antimicrobial granules can be externalized upon neutrophil activation to neutralize microbes outside the cell, termed degranulation [217]. A critical component of host defense to infection is the third response neutrophils have, the generation of neutrophil extracellular traps (NETs), which catch pathogens and prevent further migration of infection [217]. Pyroptosis, a form of programmed cell death, is then used to restrict pathogen replication and eliminate infected cells [217,218,219]. Upon completion of these responses, infected neutrophils or neutrophil debris are cleared from the infection site to prevent excessive inflammation.

The clearance of neutrophils from the infection site occurs through programmed cell death [218] and reverse migration [205,220,221]. Apoptosis, another form of programmed cell death, is used in some intracellular bacterial infections to prevent the release of intracellular contents of the infected cell through engulfment by phagocytes [218]. Rather than using programmed cell death, other infected neutrophils can reverse migrate. Reverse migration is the process of activated neutrophils leaving the infection site to return into circulation, and was first visualized in zebrafish, further highlighting the power of this aquatic model. While research has shown that reverse migration occurs, the process remains poorly understood. Degranulation and the production of NETs end with the pathogen neutralized and preventing dissemination; however, the pathogen has not been killed or cleared. To clear the remains of degranulated neutrophils or NETs, additional phagocytic cells are necessary to engulf the debris.

#### 5.3.2. Macrophages

Although neutrophils eliminate pathogens in the infection site, they also recruit macrophages, which are the primary cell type responsible for phagocytosis of the pathogen and neutrophil debris in response to infection [222,223]. Macrophages are known to have at least two different presentations: proinflammatory (formerly known as M1) and anti-inflammatory (M2) [223]. Recent single-cell transcriptomic experiments now suggest that there is a spectrum of macrophage states [224]. How macrophages transition between these states in response to a variety of insults is an active area of investigation. Proinflammatory macrophages have antimicrobial properties and respond to bacterial infection [110,225]. These macrophages will migrate to the infection site in response to cytokines and phagocytose the microbe killing the pathogen [211,226]. Macrophages have additional tasks they complete while in the site of infection than just phagocytosis, as diagramed in Figure 3. One task is a form of intercellular exchange named shuttling. In this process, a unidirectional transfer of the pathogen occurs from a neutrophil to a macrophage through phagosome transfer [226]. This process is both beneficial and harmful as the transfer allows for the macrophage to kill the majority of pathogens; however, some pathogens manipulate this process to further spread in the host [226]. The formation of granulomas is another task macrophages have in response to some infections. Granulomas, found in TB infections, are a defense mechanism to cluster infected macrophages and bacteria together and shield surrounding tissues from the infection [104,111]. The formation of granulomas, the process of unidirectional shuttling and phagocytosis make macrophages the final step of the innate immune response in the clearance of infection.

#### 5.3.3. Tissue Resident

Some tissues have an additional form of innate immune protection through tissue-resident macrophages. Tissue-resident macrophages act as local surveillance [227], coordinate the inflammatory response [227], and assist with pathogen containment [228]. The main tissue-resident cell population studied is in the central nervous system (CNS). Microglia are the tissue-resident macrophages in the CNS, and in adult zebrafish there are two populations of microglia matching that of human microglia [229]. Microglia function is conserved in larval zebrafish [230,231]. The conservation of microglia between humans and larval zebrafish allows for time lapse imaging of the microglial response to CNS infection and the integration of peripheral immune cells in pathogen response. In addition to the CNS, the liver, heart, and gut also have resident macrophages [232]. Both the liver and heart-resident macrophages act as front line defenders for circulation. The gut has specialized lysosome-rich enterocytes in combination with resident macrophages, which trap bacteria in the gut, preventing bacterial translocation into the fish [233]. Together, these tissue-resident macrophage populations demonstrate that the innate immune defense in zebrafish is organized locally as well as systemically, with resident cells acting as first-line sentinels that coordinate the entry of peripheral immune cells when local containment is insufficient. This makes zebrafish especially useful for visualizing how tissue-specific macrophage populations interact with pathogens in real time within an intact vertebrate host.

### 5.4. Neutrophil and Macrophage Vulnerability

Despite the strong response from neutrophils and macrophages, like barriers, these defenses can also fail. For example, in mammals, neutrophils infected with the bacterial pathogen *Porphyromonas gingivalis* can be captured alive by macrophages resulting in transcriptional reprogramming of macrophages [234]. Whether this is a common mechanism exploited by aquatic pathogens is still unknown. Further, granulomas can also be used by bacteria as a “safe space” for replication and persistence due to granulomas preventing further immune response to the surviving bacteria [235,236]. This vulnerability is observed across aquatic hosts as *Piscirickettsia salmonis* can replicate within salmonid macrophages [237]. Interestingly, some species, such as *V. anguillarum*, have developed mechanisms to support intracellular survival without exploiting granulomas. Both tissue-resident macrophages and systemic macrophages can be evaded or manipulated by specific pathogens. In addition, host macrophages can be hijacked by the pathogen (e.g., *Burkholderia cenocepacia*) to increase spread and harm to the host while preventing pathogen death [99]. In proinflammatory macrophages, fungal pathogens are capable of colonizing macrophages, allowing for secondary fungemia and lack of clearance, with the fungus using the macrophage as a trojan horse to spread [238,239]. The bacteria *M. marinum* causes polarization changes in macrophages toward an anti-inflammatory M2 phenotype allowing for prolonged survival in the host shown in zebrafish macrophages [240,241]. In an attempt to prevent further manipulation, macrophages use a regulated form of necrotic cell death termed necroptosis [218,242]. Uncontrolled release of intracellular contents provokes further inflammation and requires additional macrophages to respond for clearance. Necroptosis has also been observed in other teleost fish [243] as well as mammals [244], highlighting the importance of this mechanism of cell death for host protection. However, many questions still remain including the following: (1) what mediates the switch between necroptosis being beneficial versus detrimental to the host; (2) identifying the mechanism(s) that pathogens use to manipulate necroptotic signaling, and (3) whether this mechanism can affect cells of the adaptive immune system. Aquatic models offer an advantage to tease apart some of these outstanding questions.

### 5.5. Use of Infection Niches to Observe and Perturb Interaction Outcomes

Pathogens can persist within specific tissues and cells, even after clearance of systemic infection. Therefore, aquatic host–pathogen research has begun to develop ways to visualize and probe infection niches [237,245,246,247,248,249]. As zebrafish are a well-conserved model for studying immune biology, the community has developed different infection methods to test tissue specific infections and compare them with systemic infections through various injection locations (Table 2). Zebrafish are ideally suited for these types of studies because the injection site can be selected to model either systemic or localized infection while allowing live imaging of host–pathogen interactions.

Injection into the caudal vein/blood island [250,251,252], yolk body [253,254], or Duct of Cuvier [250,255] will cause a rapid, systemic infection. This method has been used to model *Salmonella typhimurium*, *P. aeruginosa*, and *M. marinum* [250,252]. Localized injection methods have been developed for swim bladder [256,257,258], otic vesicle [259,260,261], hindbrain ventricle [262,263], notochord [264], and pericardial cavity [265]. The role of infection niches in determining outcome to exposure is highlighted by work with the fungus *Mucor circinelloides*. Injection of spores into the swim bladder led to a weak inflammatory response, whereas injection into the hindbrain ventricle led to immune cell recruitment within 24 h [263]. Together, these studies demonstrate that zebrafish injection site choice can be matched to the biological question, with vascular sites most appropriate for studying systemic infection and localized injection best suited for studying immune cell trafficking, infection niches, tissue-specific inflammation, and real-time host–pathogen dynamics.

**Table 2:** **Examples of tissue-specific infection models in** **zebrafish.**

Injection Site	Modeled Region	Pathogen	References
**Systemic**			
Caudal vein/blood island	Systemic	*S. typhimurium*	[222,250]
		*M. marinum*	[250,251,266]
		*E. tarda*	[267]
Duct of Cuvier	Systemic	*S. typhimurium*, *M. marinum*	[250]
		*T. cruzi*	[255]
		*S. aureus*	[268]
		*P. aeruginosa*	[269]
Yolk	Systemic	*S. agalactiae*	[254]
		*C. turicensis*	[253]
		*T. cruzi*	[270]
Immersion	Systemic	*P. aeruginosa*	[271,272]
		*V. fischeri*	[140]
		*E. tarda*	[267]
		*V. parahaemolyticus*	[273]
		*V. anguillarum*	[274]
**Niche**			
Hindbrain ventricle	Central nervous system	*M. marinum*	[266,275]
		*A.* *fumigatus*	[276]
		*M. circinelloides*	[263]
Otic vesicle	Inner ear	*K. pneumoniae*	[259]
		*S. iniae*	[260,261]
Notochord	Bone/cartilage	*E. coli*	[98,264]
		*M. marinum*	[277]
Pericardial cavity	Heart	*S. aureus*	[268]
		Rift Valley fever virus	[265]
		*E. coli*	[278]
Swim bladder	Epithelial/Lung	*C. albicans*	[257]
		*K. pneumoniae*	[279]
		*M. circinelloides*	[263]

## 6. Remaining Challenges in Aquatic Host–Pathogen Research

Aquatic systems are powerful models used to study host–pathogen interactions, but interpretation of results can depend on species, developmental stage, route of infection, availability of tools, and the pathogen. As such, aquatic host–pathogen interaction studies should focus on utilizing the strength of the model to their advantage. For example, as discussed, many aquatic species lack an adaptive immune system or do not develop one until later in development. This can be advantageous when studying innate immunity, but limiting if interested in vaccine responses or immune memory, which take longer and require the adaptive immune system. For these studies, *Xenopus* may provide a better option over the highly utilized zebrafish. Similarly, injection of pathogens can create an artificial niche. Further, injections bypass the physical and physiological barriers aquatic species use as a natural defense. As such, alternative methods of infection such as bath application [280] and feeding [281] are used to better understand the interaction between pathogens and skin, gills, and mucosal surfaces. Lastly, the aquatic environment adds additional variability to experiments. In the wild, aquatic species face changing water chemistry, temperature, oxygenation, and stress; however, all these variables can influence immunity and pathogen behavior. Comparing across multiple aquatic models or types of pathogenic exposure will provide new insights into these complex associations. Therefore, experimental design when choosing models and environmental conditions should be considered as part of the biological framework.

## 7. Conclusions

Aquatic host–microbe systems provide a powerful framework for understanding how infection outcomes emerge from dynamic interactions among hosts, microbes, and the environment. Across a range of invertebrate and vertebrate models, ranging from sea anemones and brine shrimp to zebrafish and *Xenopus*, aquatic organisms reveal that disease is not solely determined by the presence of a pathogen or the susceptibility of a host. Instead, infection outcomes arise from the interplay between microbial traits, host defenses, and environmental conditions. The *Vibrio* genus exemplifies this complexity since closely related species can occupy mutualism, commensalism, and pathogenesis roles. The shared molecular toolkit used by both pathogens and symbionts further challenges the traditional binary classification and highlights the importance of conditional pathogenicity, host-dependent responses, and environmentally regulated virulence.

Aquatic models provide critical insights into the mechanisms that shape pathogenicity outcomings, including physical and physiological barriers, microbiome-mediated protection, innate immune responses, and tissue-specific infection niches. Among aquatic vertebrates, zebrafish have emerged as a particularly influential model because they combine physiological complexity, genetic tractability, and translational relevance that enables mechanistic investigation of host–pathogen interactions across multiple biological scales. Together, these aquatic host–microbe systems demonstrate that microbial behavior and host susceptibility are highly context-dependent, with neither existing as a fixed biological state. Future studies integrating multiple aquatic models, environmental variables, advanced imaging, and genetic approaches will be essential for uncovering the principles that govern host–microbe interactions across biological systems.

## Figures and Tables

**Table 1:** ***Vibrio* species discussed in this review, their hosts, and documented** **role.**

Species	Host(s)	Documented Role
*Vibrio cholerae*	Human, fish, shrimp	Pathogen, strain-dependent
*Vibrio vulnificus*	Human, shellfish, fish	Pathogen, host-health-dependent
*Vibrio parahaemolyticus*	Human, shrimp, bivalves, fish	Pathogen, strain-dependent
*Vibrio coralliilyticus*	Coral, sea anemone, oyster, brine shrimp	Pathogen
*Vibrio harveyi*	Oyster, shrimp, crabs, fish	Pathogen
*Vibrio proteolyticus*	Coral, brine shrimp, fish	Pathogen
*Vibrio* (*Aliivibrio*) *fischeri*	*Euprymna* squid, brine shrimp, zebrafish	Mutualist, host-dependent
*Vibrio* (*Aliivibrio*) *logei*	*Sepiola* squid	Mutualist

## Data Availability

No new data were created or analyzed in this study. Data sharing is not applicable to this article.

## Abbreviations

The following abbreviations are used in this manuscript:

TLR	toll-like receptor
BBB	blood–brain barrier
CSF	cerebral spinal fluid
dpf	days post-fertilization
NETs	neutrophil extracellular traps
CNS	central nervous system
T3SS	Type III Secretion System
LPS	Lipopolysaccharide
T6SS	Type VI Secretion System
